# Financial Impacts of the COVID-19 Pandemic on U.S. Older Adults: Assessing Pandemic-induced Job and Income Loss

**DOI:** 10.1093/geroni/igab046.3606

**Published:** 2021-12-17

**Authors:** Walter Dawson, Nora Mattek, Sarah Gothard, Jeffrey Kaye

**Affiliations:** 1 Oregon Health & Science University and Global Brain Health Insitute, Portland, Oregon, United States; 2 Oregon Health and Science University, Portland, Oregon, United States; 3 Oregon Health & Science University, Portland, Oregon, United States

## Abstract

The COVID-19 pandemic has greatly impacted the economic security of millions of older adults. Job loss and reductions in personal income were significant in 2020 stemming from pandemic-induced shutdowns that temporarily closed large swaths of the U.S. economy. Yet, the specific financial impacts of the pandemic on older adults, including family care partners, are not well understood. To understand the COVID-19 pandemic’s effects on the health and financial well-being of older adults, we gathered data from the Research via Internet of Technology and Experience (RITE) Study, a longitudinal survey panel providing data from thousands of participants of various ages and backgrounds in the U.S. on their use of healthcare and technology (N=1,365). We measured by population strata including age, sex, and education and other characteristics including caregiver status. Adults between 20-40 years of age experienced the highest rate of job loss and reduction in wages (33%) as a result of the pandemic, while adults aged >70 years experienced the lowest rate (12.5%). However, adults aged 50-60 and 60-70 also experienced relatively high levels of job loss at (28.4% and 25.7%, respectively). Behavior changes and disruptions to typical routines to avoid COVID-19 infections may have contributed to job and personal income loss amongst Individuals aged 50-60 and 60-70. However, these findings suggest potentially high levels of economic insecurity amongst individuals who continue to work into late-life. These results may help policymakers understand how to better tailor interventions and policies to mitigate economic insecurity, particularly for populations disproportionately impacted by the pandemic.

